# Supplementary material for: Obesity-Related Complications Including Dysglycemia Based on 1-h Post-Load Plasma Glucose in Children and Adolescents Screened before and after COVID-19 Pandemic
Source: Nutrients. 2024 Aug 5;16(15):2568. doi: 10.3390/nu16152568 (PMC11314267; doi:10.3390/nu16152568)
Supplement: Supplementary file 1 [file nutrients-16-02568-s001.zip › nutrients-3132990-supplementary.pdf]

Obesity-related complications including dysglycemia based on 1-hour post-load plasma glucose in children and adolescents screened before and after COVID-19 pandemic

Supplementary materials

Table S1. Results of OGTT in the whole study group and in the subgroups diagnosed before and after COVID-19 pandemic (PRE and POST)

	All	PRE	POST	p
Glucose 0 (FPG) [mg/dl]	84 (79-90)	82 (77-88)	86 (81-91)	**<0.001**
Glucose 0.5h-PG [mg/dl]	143 (128-159)	143 (125-161)	142 (132-157)	0.909
Glucose 1h-PG [mg/dl]	140 (120-162)	134 (113-159)	144 (122-168)	**0.024**
Glucose 1.5h-PG [mg/dl]	124 (112-141)	123 (110-141)	124.5 (112.5-141.5)	0.477
Glucose 2h-PG [mg/dl]	118 (104-134)	116 (103-133)	118 (104-134)	0.495
Insulin 0 [mIU/l]	16.0 (10.6-21.6)	18.0 (12.0-23.7)	13.4 (10.1-19.1)	**<0.001**
Insulin 0.5h-PG [mIU/l]	101.0 (64.1-162.0)	111.0 (71.0-181.0)	94.6 (59.7-145.9)	**0.031**
Insulin 1h-PG [mIU/l]	104.0 (63.8-161.9)	104.5 (59.0-172.4)	101.9 (67.7-159.8)	0.797
Insulin 1.5h-PG [mIU/l]	93.0 (54.6-137.0)	97.0 (60.0-162.0)	90.9 (50.3-131.0)	0.152
Insulin 2h-PG [mIU/l]	75.0 (47.3-131.2)	81.0 (52.0-133.0)	72.0 (43.4-128.5)	0.200

p-values relate to the difference between groups PRE and POST in Mann-Whitney U test

Table S2. Results of OGTT in the patients with T2D, prediabetes and NGT, diagnosed according to the criteria of ADA

	NGT	Prediabetes	T2D	p
Glucose 0 (FPG) [mg/dl]	84 (79-89)	86 (81-94)	98 (90-119)	**<0.001**
Glucose 0.5h-PG [mg/dl]	140 (123-156)	146.5 (134-175)	164 (142-181)	**<0.001**
Glucose 1h-PG [mg/dl]	132 (114-150)	163.5 (136-182)	194 (162-233)	**<0.001**
Glucose 1.5h-PG [mg/dl]	119 (108-129)	142 (124-165)	204 (190-247)	**<0.001**
Glucose 2h-PG [mg/dl]	111 (100-123)	143 (127-159)	212 (192-282)	**<0.001**
Insulin 0 (fasting) [mIU/l]	14.2 (10.0-20.2)	17.0 (12.0-25.3)	17.7 (16.5-27.8)	**0.011**
Insulin 0.5h-PG [mIU/l]	87.5 (59.0-155.0)	103.8 (76.4-179.0)	87.6 (21.3-137.1)	0.253
Insulin 1h-PG [mIU/l]	101.5 (62.3-160.8)	121.0 (84.9-192.0)	72.1 (32.8-190.0)	**0.005**
Insulin 1.5h-PG [mIU/l]	76.8 (49.7-128.3)	120.9 (86.0-171.2)	62.3 (32.8-231.0)	**<0.001**
Insulin 2h-PG [mg/dl]	63.0 (40.2-95.8)	143.6 (82.0-239.0)	175.0 (47.1-246.6)	**<0.001**

p-values relate to the differences between groups T2D, prediabetes and NGT in Kruskal-Wallis test

Table S3. Results of OGTT in the groups of patients diagnosed according to the criteria of IDF

	NGT-IDF	IH-1h	Prediabetes-IDF	T2D-IDF	p
Glucose 0 (FPG) [mg/dl]	84 (78-89)	83 (81-89)	86 (80-94)	97 (86-102)	**<0.001**
Glucose 0.5h-PG [mg/dl]	135 (120-150)	155 (146-173)	145 (134-174)	180 (147-182)	**<0.001**
Glucose 1h-PG [mg/dl]	124.5 (110-140)	168 (158-177)	162 (136-178)	219 (192-226)	**<0.001**
Glucose 1.5h-PG [mg/dl]	115.5 (105-126)	127.5 (120-140)	138 (124-162)	203.5 (190-216)	**<0.001**
Glucose 2h-PG [mg/dl]	111 (101-122)	112 (99-126)	143 (125-156)	192 (161-218)	**<0.001**
Insulin 0 (fasting) [mIU/l]	15.9 (10.1-21.6)	13.0 (9.4-17.0)	17.0 (12.0-24.4)	17.7 (14.3-27.0)	**0.022**
Insulin 0.5h-PG [mIU/l]	109.3 (64.0-161.0)	85.2 (60.3-151.0)	101.5 (76.4-179.0)	90.6 (41.6-141.0)	0.418
Insulin 1h-PG [mIU/l]	81.2 (54.0-131.0)	134.7 (82.9-225.4)	120.2 (84.9-192.0)	177.6 (58.0-217.9)	**<0.001**
Insulin 1.5hPG [mIU/l]	75.0 (48.8-129.3)	103.4 (62.5-127.2)	120.0 (84.9-170.0)	129.2 (34.9-203.7)	**<0.001**
Insulin 2h-PG [mg/dl]	67.4 (43.7-98.9)	52.0 (33.6-82.0)	140.2 (79.0-239.0)	175.0 (47.1-246.4)	**<0.001**

p-values relate to the differences between the groups in Kruskal-Wallis test
